# Associations between maternal serum HDL-c concentrations during pregnancy and neonatal birth weight: a population-based cohort study

**DOI:** 10.1186/s12944-020-01264-0

**Published:** 2020-05-14

**Authors:** Hongliang Wang, Qinyu Dang, Haiyan Zhu, Ning Liang, Zhiyin Le, Dongxu Huang, Rong Xiao, Huanling Yu

**Affiliations:** 1 School of Public Health, Capital Medical University, Beijing, 100069 China; 2 Fuxing Hospital, Capital Medical University, Beijing, 100053 China

**Keywords:** High-density lipoprotein cholesterol, Pregnancy, Birth weight, Small for gestational age

## Abstract

**Background:**

To evaluate the associations between maternal serum concentrations of high-density lipoprotein cholesterol (HDL-c) throughout pregnancy and neonatal birth weight (BW) and small for gestational age (SGA) births.

**Methods:**

A prospective cohort of 2241 pregnant women was followed from recruitment to delivery in three hospitals in Beijing, China between January 2014 and December 2017. Maternal fasting serum lipids concentrations were measured at gestational week 6–12, 16, 24 and 36. Major outcome was neonatal BW. The associations between maternal HDL-c and BW were estimated by linear regression and linear mixed-effects models. Odds ratios (ORs) and 95% confidence intervals of SGA births in relation to HDL-c were evaluated via logistic regression analysis.

**Results:**

There was a tendency that mothers with higher HDL-c concentrations throughout gestation gave birth to infants with lower BW. A negative association was found between maternal HDL-c concentrations and BW at 24th and 36th gestational weeks (B = − 34.044, *P* = 0.034; B = − 53.528, *P* = 0.000). The HDL-c trend of change was inversely associated with BW (B = − 442.736, *P* = 0.000). Mothers with SGA neonates had higher serum HDL-c concentration at the 36th gestational week (*P* < 0.01). The incidences of SGA in the three groups (HDL-c: 1.84–2.23 mmol/L, 2.24–2.59 mmol/L and ≥ 2.60 mmol/L) were higher than the group with the lowest concentration of HDL-c (< 1.83 mmol/L) (*P* < 0.01, *P* < 0.01, *P* < 0.001) at 36th week. Higher maternal HDL-c concentrations at 36th week (HDL-c: 1.84–2.23 mmol/L, 2.24–2.59 mmol/L and ≥ 2.60 mmol/L) were positively associated with the incidence of SGA (OR = 1.900, *P* = 0.008; OR = 1.893, *P* = 0.008; OR = 1.975, *P* = 0.004). The HDL-c trend of change was positively associated with SGA births (OR = 9.772, *P* = 0.000).

**Conclusions:**

Maternal serum HDL-c concentrations were inversely associated with BW at 24th and 36th gestational weeks. The high concentrations of HDL-c at the 36th gestational week increased the risk of SGA. The maternal HDL-c trend of change across pregnancy was associated with smaller neonatal size.

## Background

Maternal lipid levels throughout gestation increase as part of a physiological response to pregnancy [1]. HDL-c concentration changes non-significantly in the first trimester, but significantly increases in the second trimester and slightly decreases in the third trimester [2]. These changes are considered to contribute to fetal growth and development. Nevertheless, high HDL has been linked to smaller neonatal size [3]. In contrast, relative low HDL-c concentration across pregnancy was substantially associated with an increased risk for macrosomia [4].

Inappropriate fetal growth, which is often assessed by birth weight (BW), has been in relation to adverse pregnancy outcomes and chronic diseases in adulthood [3, 5]. Small for gestational age (SGA) is calculated by BW [6]. Previous studies have shown associations between HDL and BW or SGA. Misra et al. [7] found that aBW (adjusted for gestational age at delivery) was negatively associated with HDL-c at any time point starting at 10th gestational week in overweight/obese women. The mean concentrations of total HDL particles were significantly higher in the mothers with SGA infants compared with the controls [5]. SGA and low birth weight (LBW) have been associated with increased risks for adverse cardiometabolic outcomes in both mother and offspring in later life. LBW/SGA of the offspring may indicate increased maternal cardiovascular disease risk [8, 9]. There are already risk factors of developing type 2 diabetes mellitus (DM) and cardiovascular disease (CVD) during childhood in short prepubertal children born SGA [10]. However, Azadbakht et al. [11] found that the risk for high diastolic blood pressure (DBP) of LBW adolescents was lower than that of normal BW adolescents.

The association between HDL-c concentration measured at one or two time points during gestation and BW has been studied, but the conclusions were inconsistent [12, 13]. Especially the odds ratio (OR) of HDL-c for SGA was not determined. A prospective cohort of pregnant women was constructed in this study to investigate the associations between maternal HDL-c concentrations and BW. Maternal serum HDL-c concentrations were measured at four time points during pregnancy and BW was recorded after delivery. In addition, this study also evaluated whether the concentration of HDL-c was a risk factor for SGA, as well as the relationships between HDL-c changes throughout gestation and BW or SGA, since there are only a few studies which have observed the associations between HDL-c longitudinal change and pregnancy outcomes.

## Methods

### Study population

A cohort study was conducted in pregnant women who attended prenatal care at Fuxing Hospital affiliated to Capital Medical University, Xuanwu Hospital affiliated to Capital Medical University and Maternal and Child Health Hospital of Fengtai District, China from January 2014 to December 2017. Inclusion criteria were: 1) singleton pregnancy; 2) naturally conceived. Exclusion criteria were: 1) women with pre-pregnancy CVD, known DM, gestational diabetes mellitus (GDM), chronic hypertension or preeclampsia (PE); 2) maternal age < 20 years and > 40 years; 3) fetuses diagnosed with congenital malformation or APGAR scores < 7. Of the total of 3749 pregnant women recruited in this study, 642 women with GDM, 130 women with preterm births (gestational week < 37) and 410 women with gestational week > 40 were excluded. In addition, 294 mothers were excluded for the information of HDL-c concentration missing and 32 mothers/babies were excluded for other information missing (including pre-pregnancy BMI (pre-BMI), parity, BW and etc.). Finally, 2241 pregnant women were eligible for the study. They were followed from recruitment to delivery. There were 1676 cases in Fuxing Hospital, 192 cases in Xuanwu Hospital and 409 cases in Maternal and Child Health Hospital of Fengtai District. This study was approved by the Ethics Committee of Capital Medical University and all participants have signed the informed consent after being fully informed at the first visit.

### Covariates

The information of demographic and maternal characteristics, including age, pre-pregnancy weight and height (pre-BMI calculated, kg/m^2^), parity, blood pressure and history of disease (CVD, DM, chronic hypertension and etc.), was collected by questionnaire. According to China cutoff points, pregnant women were classified as underweight (BMI <  18.5 kg/m^2^), normal weight (18.5 kg/m^2^ ≤ BMI < 24.0 kg/m^2^) or overweight/obese (BMI ≥ 24.0 kg/m^2^). Gestational weight gain (GWG) was calculated as the difference between the body weight at delivery and self-reported pre-pregnancy weight. The diagnosis of GDM was made by the criterion of Association of Diabetes and Pregnancy Study Groups (IADPSG): one or more of the following values is met or exceeded in the 75-g Oral Glucose Tolerance Test (OGTT): fasting glucose, 5.10 mmol/L; 1-h glucose, 10.00 mmol/L; 2-h glucose, 8.50 mmol/L.

### Outcomes

The infants’ characteristics, including sex, BW, body length, gestational age, delivery mode and perinatal outcomes, were collected at delivery. Gestational age was determined by combination of the last menstrual period and the early first trimester ultrasound. Neonates were classified into SGA, appropriate for gestational age (AGA) and large for gestational age (LGA) based on BW for gestational age. SGA was defined as BW below the 10th percentile and AGA was defined as BW between the 10th and the 90th percentile.

### Lipid measurement

Fasting blood samples were collected at gestational week 6–12, 16, 24 and 36. The serum concentrations of total cholesterol (TC), triglycerides (TG), low-density lipoprotein cholesterol (LDL-c) and HDL-c were measured by the Hitachi type 7180 automatic biochemical analyzer (Hitachi High-Tech Science Systems Corporation, Yokohama, Japan) at the laboratory of Fuxing Hospital, individually with the Unicel 36 DX600 Synchron Clinical System (Beckman-Coulter, Mississauga, ON, Canada) at the laboratory of Xuanwu Hospital and using the Dimension EXL with LM automatic biochemical analyzer (Siemens Healthcare Diagnostics, Shanghai, China) at the laboratory of Maternal and Child Health Hospital of Fengtai District.

### Statistical analyses

All data were analyzed with the SPSS 22.0 software (SPSS, Chicago, IL, USA) and Stata 15.1 (Stata Corp., College Station, TX, USA). Continuous variables were expressed as mean ± SD and categorical variables were expressed as N (%). Pearson correlation and multiple liner regression analyses were carried out to analyze the associations between maternal HDL-c concentrations and BW. A two-stage approach [14] was carried out to analyze the associations between HDL-c trend of change during pregnancy and BW and SGA. In the first stage, a linear mixed-effect model (LME) was constructed for HDL-c as a function of gestational week at sampling and predicted the best linear unbiased predictor (BLUP) of random intercept and slope. The predicted intercept represents the mean HDL-c concentration at the 6-12th gestational week. The predicted slope represents the trend of HDL-c concentration changes throughout gestation. Secondly, the BLUP estimates of intercept and slope were used as predictors in the linear and logistic regression models including BW and SGA as the outcomes, respectively. The odds ratios (ORs) and 95% confidence intervals (95% CIs) of SGA births in association with HDL-c concentrations or HDL trend of change were evaluated using logistic regression analysis. *P* < 0.05 indicated statistically significant.

## Results

### Characteristics of study population

Table 1 presents the characteristics of pregnant women and neonates. The mean maternal age was 30.331 ± 3.670 years old. The mean maternal pre-BMI was 21.622 ± 2.954 kg/m^2^. About 17.7% of the participants were stratified as overweight/obese for pre-BMI ≥ 24.0 kg/m^2^. The mean GWG between the first visit and the fourth visit was 13.374 ± 4.118 kg. The mean gestational age at birth was 39.272 ± 0.873 weeks and the mean BW was 3382.644 ± 386.404 g.

**Table 1:** Characteristics of pregnant women and neonates

Characteristics	Mean ± SD or N (%)
**Maternal characteristics**	
Age (years)	30.331 ± 3.670
20–29	1019 (45.50)
30–34	896 (40.00)
35–40	326 (14.50)
Pre-pregnancy BMI (kg/m^2^)	21.622 ± 2.954
< 18.5	260 (11.60)
18.5–23.9	1585 (70.70)
≥ 24	396 (17.70)
Gestational weight gain (kg)	13.374 ± 4.118
Parity	
1	1482 (66.10)
> 1	759 (33.90)
**Neonatal characteristics**	
Sex	
male	1177 (52.50)
female	1064 (47.50)
Gestational age (weeks)	39.272 ± 0.873
Birth weight (g)	3382.644 ± 386.404
< 2500	15 (0.70)
2500–3999	2087 (93.10)
≥ 4000	139 (6.20)
Weight for gestational age	
SGA	204 (9.10)
AGA	1821 (81.30)

*SGA* small for gestational age, *AGA* appropriate for gestational age

### Associations between maternal HDL-c throughout pregnancy and neonatal birth weight

**Fig. 1:**
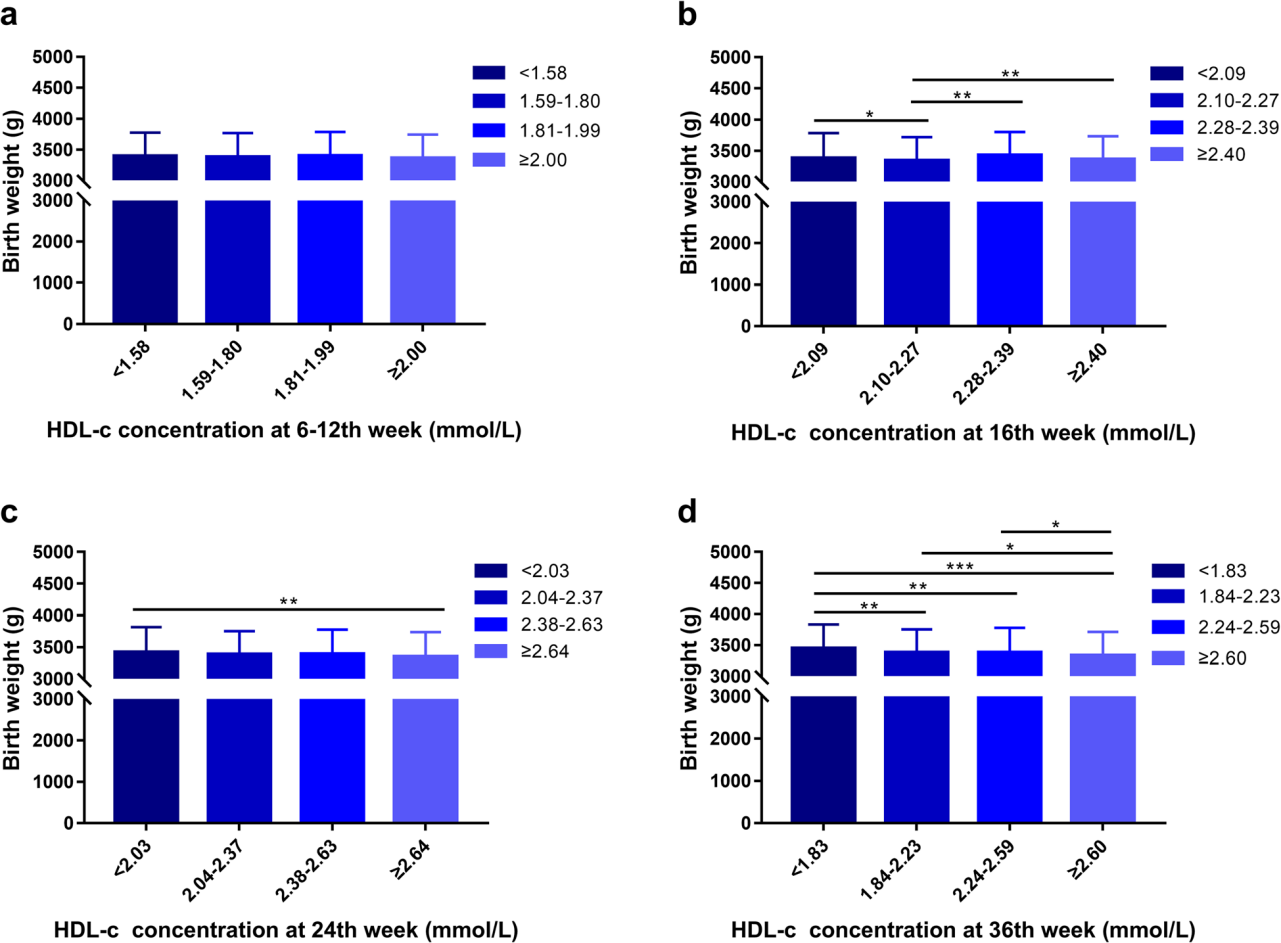
HDL-c concentration and mean (± SD) birth weight. **a** 6-12th week. **b** 16th week. **c** 24th week. **d** 36th week. ^*^P < 0.05, ^**^P < 0.01, ^***^P < 0.001

There was a tendency that pregnant women with higher HDL-c concentrations throughout pregnancy gave birth to infants with lower BW (Fig. 1). A negative association between maternal HDL-c concentrations and BW at 24th and 36th gestational weeks was found by Pearson correlation analysis (*P* < 0.01, *P* < 0.001) (Table 2).

**Table 2:** Pearson correlations between maternal HDL-c concentrations throughout pregnancy and birth weight

	6-12th week	16th week	24th week	36th week
r	−0.020	−0.005	− 0.067^**^	−0.100^***^
*P* value	0.355	0.829	0.002	0.000

^**^P < 0.01, ^***^P < 0.001

The associations between maternal HDL-c concentrations and neonatal BW at the four time point measurements throughout gestation were evaluated by multiple liner regression analyses. Maternal concentrations of HDL-c were inversely associated with BW at 24th and 36th gestational weeks (B = − 34.044, *P* = 0.034; B = − 53.528, *P* = 0.000). No significant association between maternal HDL-c concentrations and BW was found at 6-12th or 16th week. The variable of age was negatively associated with BW at the 6-12th week and 16th week. Other variables, including pre-BMI, GWG and gestational age were positively associated with BW throughout gestation. (Table 3).

**Table 3:** The associations between maternal HDL-c concentrations and birth weight

	6-12th week		16th week		24th week		36th week	
	B	*P* value	B	*P* value	B	*P* value	B	*P* value
HDL-c	31.295	0.171	25.158	0.256	−34.044	0.034	−53.528	0.000
Age	−4.569	0.042	−4.441	0.048	−3.796	0.092	−3.380	0.132
Pre-BMI	29.689	0.000	29.457	0.000	28.291	0.000	28.149	0.000
Parity	115.869	0.000	115.146	0.000	112.544	0.000	109.712	0.000
GWG	16.323	0.000	16.315	0.000	16.632	0.000	16.368	0.000
Neonatal sex	−107.458	0.000	−107.323	0.000	− 106.146	0.000	−106.299	0.000
Gestational age	141.764	0.000	141.529	0.000	141.539	0.000	141.767	0.000

The model was adjusted for maternal age, pre-BMI, parity, GWG, neonatal sex and gestational age

*Pre-BMI* pre-pregnancy BMI, *GWG* gestational weight gain

The association between HDL-c trend of change throughout pregnancy and BW was evaluated by LME and liner regression model. Maternal HDL-c trend of change across pregnancy was inversely associated with BW (B = − 442.736, *P* = 0.000) (Table 4).

**Table 4:** The association between maternal HDL-c trend of change and birth weight

	B	*P* value
HDL-c		
Intercept	98.111	0.000
Slope	−442.736	0.000
Age	−4.139	0.000
Pre-BMI	29.270	0.000
Parity	122.306	0.000
GWG	16.494	0.000
Neonatal sex	−106.412	0.000
Gestational age	142.868	0.000

The model was adjusted for maternal age, pre-BMI, parity, GWG, neonatal sex and gestational age

*Pre-BMI* pre-pregnancy BMI, *GWG* gestational weight gain

### Association between maternal HDL-c throughout pregnancy and SGA

**Fig. 2:**
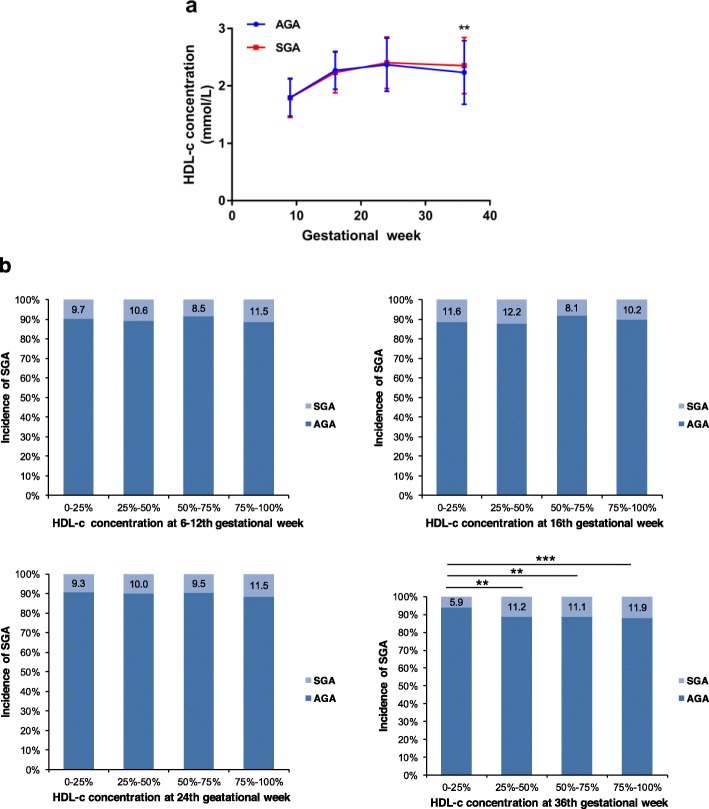
**a** Mean (± SD) HDL-c concentrations between two groups during pregnancy. **b** The incidence of SGA of different maternal HDL-c concentrations during pregnancy. AGA = appropriate for gestational age; SGA = small for gestational age. ^**^P value < 0.01, ^***^P value < 0.001

There was a tendency that mothers with SGA infants had higher serum HDL-c concentration. The analysis of longitudinal trend of HDL-c concentrations throughout pregnancy manifested that HDL-c concentration of SGA group was higher than AGA group at the 36th week (Fig. 2a). In addition, there was a tendency that the incidence of SGA consistently increased with the increasing of HDL-c concentrations at the 24th and 36th weeks. The incidences of SGA in the three groups (HDL-c: 1.84–2.23 mmol/L, 2.24–2.59 mmol/L and ≥ 2.60 mmol/L) were higher than the group with the lowest concentration of HDL-c (< 1.83 mmol/L) (*P* < 0.01, *P* < 0.01, *P* < 0.001) at 36th week (Fig. 2b).

Higher maternal HDL-c concentrations at 36th week (HDL-c: 1.84–2.23 mmol/L, 2.24–2.59 mmol/L and ≥ 2.60 mmol/L) were positively associated with the incidence of SGA (OR = 1.900, *P* = 0.008; OR = 1.893, *P* = 0.008; OR = 1.975, *P* = 0.004). Maternal GWG, pre-BMI (< 18.5 and ≥ 24) and parity > 1, were positively associated with the incidence of SGA during pregnancy. (Table 5).

**Table 5:** The associations between maternal HDL-c concentrations and risk of SGA

		6-12th week			16th week			24th week			36th week		
		OR	95% CI	*P*	OR	95% CI	*P*	OR	95% CI	*P*	OR	95% CI	*P*
HDL-c	G1	Reference			Reference			Reference			Reference		
	G2	1.000	0.653–1.531	1.000	0.998	0.629–1.582	0.992	1.098	0.711–1.696	0.674	1.900	1.185–3.044	0.008
	G3	0.839	0.544–1.292	0.425	0.690	0.466–1.022	0.064	0.977	0.641–1.491	0.916	1.893	1.182–3.033	0.008
	G4	1.050	0.698–1.579	0.815	0.810	0.546–1.203	0.297	1.195	0.788–1.811	1.195	1.975	1.242–3.139	0.004
Age		1.019	0.973–1.067	0.423	1.018	0.972–1.067	0.450	1.019	0.973–1.067	0.421	1.015	0.970–1.054	0.515
GWG		0.957	0.921–0.993	0.021	0.959	0.924–0.996	0.032	0.955	0.920–0.992	0.018	0.955	0.920–0.992	0.018
Pre-BMI	18.5–23.9	Reference			Reference			Reference			Reference		
	< 18.5	1.520	1.024–2.255	0.038	1.560	1.050–2.317	0.028	1.500	1.011–2.225	0.044	1.542	1.039–2.290	0.032
	≥ 24	0.423	0.250–0.719	0.001	0.417	0.246–0.707	0.001	0.422	0.249–0.713	0.001	0.433	0.256–0.733	0.002
Parity	1	Reference			Reference			Reference			Reference		
	> 1	0.468	0.316–0.694	0.000	0.475	0.320–0.705	0.000	0.461	0.311–0.684	0.000	0.483	0.325–0.717	0.000

The model was adjusted for maternal age, GWG, pre-BMI and parity

*SGA* small for gestational age, *pre-BMI* pre-pregnancy BMI, *GWG* gestational weight gain

The association between HDL-c trend of change across pregnancy and SGA was evaluated by LME and logistic regression model. Maternal HDL-c trend of change throughout gestation was positively associated with the incidence of SGA (OR = 9.772, *P* = 0.000) (Table 6).

**Table 6:** The association between maternal HDL-c trend of change and risk of SGA

	OR	95% CI	*P* value
HDL-c			
Intercept	0.563	0.425–0.747	0.000
Slope	9.772	3.396–28.124	0.000
Age	1.020	0.997–1.044	0.090
GWG	0.966	0.948–0.983	0.000
Pre-BMI			
18.5–23.9	Reference		0.000
< 18.5	1.543	1.266–1.879	0.000
≥ 24	0.406	0.311–0.529	0.000
Parity			
1	Reference		0.000
> 1	0.467	0.383–0.568	0.000

The model was adjusted for maternal age, GWG, pre-BMI and parity

*SGA* small for gestational age, *pre-BMI* pre-pregnancy BMI, *GWG* gestational weight gain

## Discussion

In this study, a total of 2241 pregnant women were recruited, and their serum HDL-c concentrations were detected at gestational week 6–12, 16, 24 and 36. BW was collected at delivery. Multiple linear regression and linear mixed-effects models were carried out to observe the associations between HDL-c and BW. The ORs and 95% CIs of SGA births in relation to HDL-c were evaluated using logistic regression analysis. The results showed that maternal fasting serum HDL-c were inversely associated with BW at 24th and 36th gestational weeks. The high concentration of HDL-c increased the risk of SGA at the 36th gestational week. Maternal HDL-c trend of change during pregnancy was associated with smaller neonatal size.

During pregnancy, trophoblasts absorb glycerol and free fatty acids released by placental enzyme-catalyzed hydrolysis of HDL and LDL and re-esterify them to provide fat for fetal development [15]. HDL plays an important role in intra-follicular cholesterol homeostasis which seems to be essential for the development of embryo [16]. There was a significant increase of all maternal blood lipids during pregnancy, including HDL-c [17]. However, metabolic disorder of lipids has been linked to poor pregnancy outcomes and offspring health in childhood. Hypercholesterolemia is associated with preterm delivery/LBW in term infants [18]. An increase in TC concentrations in early pregnancy was related with greater skinfold thickness (95%CI: 1.41, 5.20) of offspring at 4 years of age [19]. Maternal metabolic disorder and intrauterine conditions disturbance affect fetal metabolism, and maternal TC and TG were related to offspring adiposity [20]. Elevated level of LDL-c was associated with an increased risk for preterm birth (PTB) [21]. In addition, Romundstad et al. [9] found that women with unfavorable levels of lipid tend to give birth to large babies.

In physiological state, HDL protects against cardiovascular disease via its anti-oxidative and anti-inflammatory properties [22]. However, in diseases states, normal HDL converts to dysfunctional HDL which regulates vascular endothelial cell function differently [23]. Several studies have reported that the concentrations of HDL-c were related to pregnancy outcomes and complications. There was a significant correlation between elevated HDL-c and apoA1 concentrations and the increased odds of spontaneous preterm delivery (SPTD) [24]. Boghossian et al. [3] found that maternal HDL which failed to decrease throughout gestation was associated with smaller neonatal size. The mean concentrations of total HDL particles, medium and small HDL particles were higher in mothers with SGA infants [5]. What’s more, relative low maternal HDL-c throughout pregnancy was associated with increased risks for macrosomia and GDM [4]. HDL_2_-c level was lower in SGA neonates [25].

Several studies examined the concentration of HDL-c measured at one time point during pregnancy and observed its association with BW. Maternal fasting HDL-c was a predictor of offspring’s BW in gestational week 28 [12]. There was a tendency that mothers with higher HDL concentrations during pregnancy gave birth to infants with lower BW, especially at the second and third trimesters in this study. Muud et al. [1] found that in overweight/obese women, high HDL-c was negatively (*β* = − 0.29, 95% CI: − 0.54 to − 0.04) associated with BW z-score at 16–27 weeks. In the present study, associations between HDL-c measured at four time points during pregnancy and BW were evaluated and a negative association between maternal HDL-c and BW at 24th and 36th weeks was found. In condition of PE, HDL was associated with lower BW z-scores at 33–42 weeks, and a 0.0037 mmol/L increase in HDL throughout pregnancy was associated with decreased BW z-score [3]. In this study, women with PE or other pregnancy complications and chronic diseases were excluded, and the associations between HDL-c and BW were analyzed in normal pregnancy in order to minimize the effects of diseases on BW. HDL-c was positively associated with LBW in the study of Harville et al. [26]. Nevertheless, some studies reported that HDL-c concentrations were unrelated to BW, especially in short and thin population [13, 27]. According to the research of Hwang et al. [28], no causal effect of maternal gestational HDL-c concentration on offspring BW was found. Previous study found a positive relationship between maternal pre-BMI and GWG and neonatal BW [29]. The study of Nkwabong et al. [30] showed that primiparous women were more at risk for LBW. It was reported that compared with women aged 18 to 35 years who were with parity 1 or 2, nulliparous women younger than 18 years old had the highest risk of SGA infants [31]. Considering these factors, including pre-BMI, GWG, parity and age might have effects on BW, the models of linear regression and logistic regression analyses were adjusted for them.

Kramer et al. [5] found the mean concentrations of total HDL particles were higher in the mothers with SGA infants compared with the controls at the second trimester. In the present study, mothers with SGA infants had higher HDL-c concentration at the third trimester (*P* < 0.01), and there was a tendency that the incidence of SGA consistently increased with the increasing of HDL-c concentrations at the second and third trimesters. The incidences of SGA in the three groups with higher concentrations of HDL-c were higher than the group with the lowest concentration of HDL-c at the third trimester. The results showed that, higher maternal concentrations of HDL-c at 36th week were positively associated with the incidence of SGA. When the models were additionally adjusted for hospital, the differences were not changed (data were not shown).

Considering the longitudinal design of the present study, the associations between maternal HDL-c time trend throughout gestation and BW and SGA were also evaluated. Maternal HDL-c trend of change throughout gestation was negatively associated with BW and positively associated with SGA births overall. A prospective cohort study conducted by Farias et al. [32] also reported that the change rate of gestational HDL-c was negatively associated with BW z-score. The increase in HDL was associated with decreased BW z-score in women with GDM [3]. However, Bever et al. [33] found that the increase in HDL-c concentration from pre-pregnancy to gestational week 28 was associated with a decreased risk of SGA in the BMI ≥ 25 kg/m^2^ group. This study aimed to evaluate the associations between exposure and outcomes in all participants and the models including liner and logistic regression had been adjusted for pre-BMI. When the models were additionally adjusted for hospital, the differences were not changed (data were not shown).

Previous study observed a positive association between maternal dietary fat intake and TC level in early pregnancy [17]. Exposure to a high fat diet (HFD) (control/HFD) during gestation resulted in FGR and decreased placental weight in mice [34]. In women with gestational hyperlipidemia, fetal growth and development might be affected due to the disruption of normal placental lipid transport and synthesis [35]. Infants born SGA had significantly lower levels of cord blood HDL-c [36], and it is likely that the increased maternal HDL concentrations are due to the placental dysfunction which may decrease the transport of maternal HDL across the placenta to the fetus and lead to SGA [5]. Several other mechanisms may be involved in the process of LBW/SGA. For instance, the BW was reduced in newborns of smoking pregnant women [37], and obstetric factors, placental dysfunction and numerous fetal genetic abnormalities are also involved in the process [31].

A majority of previous studies focused on TC, TG, LDL-c and their associations with pregnancy outcomes, but less attention had been paid to HDL-c. This study evaluated the associations between HDL-c concentrations and BW at four times points throughout gestation and its role in the developing of SGA, which adds to the existing studies on maternal lipids and BW. What’s more, this study has involved big samples (*n* = 2241) which might have strengthened its ability to detect associations among HDL-c and BW.

### Study limitation

However, there were still some limitations in the present study. For instance, maternal lifestyle including dietary nutrient intake and physical activity throughout gestation may be important to lipid metabolism, but enough information on diet and physical activity had not been collected. Maternal lifestyle, which could be a confounding factor, is needed to be taken into account in the relationship between maternal lipids and pregnancy outcomes in further studies.

## Conclusions

In conclusion, the results indicate that the concentration of HDL-c is important to evaluate fetal growth and the observation of gestational HDL-c changes may be used as an additional indicator of screening women at risk of delivering SGA neonates.

## Data Availability

The data that support the findings of this study are contained in the published article.

## Abbreviations

HDL-c: High-density lipoprotein cholesterol
BW: Birth weight
SGA: Small for gestational age
AGA: Appropriate for gestational age
BMI: Body mass index
GDM: Gestational diabetes mellitus
GWG: Gestational weight gain
OR: Odds ratio
95% CIs: 95% confidence intervals
SD: Standard deviation
TC: Total cholesterol
TG: Triglyceride
LDL-c: Low-density lipoprotein cholesterol
